# Assessment of the Knowledge, Attitudes, and Practices of Primary Care Physicians Toward Prediabetes in Qassim, Saudi Arabia

**DOI:** 10.7759/cureus.87454

**Published:** 2025-07-07

**Authors:** Abdullah K Alyahya, Unaib Rabbani

**Affiliations:** 1 Family Medicine Academy, Qassim Health Cluster, Buraidah, SAU

**Keywords:** attitudes, diabetes prevention, knowledge, practices, prediabetes, primary care physicians, qassim, saudi arabia

## Abstract

Background

Prediabetes is a growing public health concern globally as well as in Saudi Arabia, where early detection and intervention are crucial. Primary care physicians (PCPs) play a pivotal role in the prevention and management of prediabetes. However, gaps in knowledge, attitude, and practice (KAP) may hinder effective care delivery. This study aimed to assess the knowledge, attitudes, and practices of PCPs toward prediabetes in Qassim, Saudi Arabia, and to explore perceived barriers and potential strategies for improving its management.

Methods

A cross-sectional survey was conducted among 163 PCPs working in primary healthcare centers across Qassim province. Data were collected using a structured online questionnaire assessing the awareness of risk factors, diagnostic criteria, management approaches, screening attitudes, and practical behaviors related to prediabetes.

Results

We found that the mean knowledge, attitude, and practice scores were 3.41/5 (±0.84), 12.84/16 (±1.78), and 3.38/5 (±0.54). The mean knowledge score was significantly higher among family physicians (3.84/5) as compared to general practitioners (2.85/5), p-value <0.001. Urban physicians also scored higher (3.52/5) than their rural counterparts (2.65/5), p-value <0.001. Attitude scores demonstrated a weak but significant correlation with age (r = 0.227, p-value 0.004) and years of experience (r = 0.202, p-value 0.010), while practice scores showed a similar pattern. Gender and location of practice were not significantly associated with practice behaviors. Notably, family physicians exhibited more favorable attitudes than general practitioners (p-value 0.004). The most common barriers in prediabetes management included a lack of patient awareness (96.9%), non-compliance (96.3%), follow-up difficulties (92%), and referral issues (86.5%).

Conclusion

Although PCPs in Qassim express generally positive attitudes toward prediabetes management, knowledge and practice gaps remain, particularly among general practitioners and those practicing in rural areas. These findings underscore the need for targeted interventions, such as mandatory continuing medical education (CME), integration of prediabetes care into routine workflows, and the use of region-specific decision-support tools like red flags in the current EHR system of PHCs. Broader national studies are recommended to validate these findings and guide policy at a systemic level.

## Introduction

Prediabetes is considered a public health problem, as it is an intermediate level of glycemia and is considered a risk factor for type 2 diabetes mellitus (T2DM), cardiovascular diseases (CVDs), and other related adverse events such as amputations and blindness. Prediabetes is a condition in which there are elevated blood glucose levels, but they are below the threshold for diabetes. According to WHO criteria, prediabetes is defined as "impaired glucose tolerance (IGT) (2-h glucose 7.8-11.0 mol/L (140-199 mg/dL)) and impaired fasting glucose (IFG) (fasting glucose 6.1-6.9 mmol/L (110-125 mg/dL)) [1].

A recent study reported that in 2021, the global prevalence of IGT and IGF was 9.1% and 5.8%, respectively, which is expected to increase to 10.0% and 6.5% in 2045 [2]. This indicates a rise in the number of people with prediabetes, which will put pressure on healthcare facilities [3]. In Saudi Arabia, demographic characteristics, such as population density, change in lifestyle, alterations in diet, and urbanization, can influence the rate of prediabetes. The current Saudi population is experiencing a rise in prediabetes as the Middle East and North African region is also expected to see a rise in the number of prediabetics [1]. It is, therefore, necessary to screen for people with prediabetes and manage it appropriately to prevent the transition to T2DM, which is a debilitating disease with severe effects on the health of the affected individuals and a burden on the healthcare system [4].

Primary care physicians are the first point of call in healthcare delivery systems and have a unique opportunity and responsibility to diagnose and treat prediabetes. They play a crucial role in determining the knowledge, attitudes, and practices (KAP) that have an impact on the patients’ outcomes. Prediabetes is a condition that not only requires medical treatment but also requires changes in patient behaviors, health education, and regular check-ups. Therefore, it is crucial to evaluate the current KAP of PCPs toward prediabetes in order to determine the deficiencies [5].

Some of the knowledge that PCPs need to possess to support the screening and management of people with prediabetes includes: screening tests, diagnostic criteria, prognosis, and management. Previous studies have reported varying levels of knowledge among PCS. For example, a cross-sectional study conducted in Sudan revealed that 60.8% of the respondents had satisfactory knowledge of prediabetes [4]. Similarly, in China, 85.8% of PCPs reported they are knowledgeable about the complications of prediabetes, but there were significant knowledge deficits regarding the guidelines and criteria for diagnosing and managing prediabetes [3].

To transform knowledge into practice, it is important to have positive attitudes. This is even more relevant when it comes to screening and early management of a condition in order to take proactive measures. For example, a study conducted in Bahrain revealed that although physicians acknowledged the significance of preventive measures concerning diabetes, only 12% of them implemented measures to prevent the disease. Similarly, only 5% of the respondents recommended lifestyle management programs to the patients as the first line of treatment [5].

Some of the practices of PCPs in the management of prediabetes involve screening, diagnosis, as well as the therapeutic approach. Various practices are recommended for the identification and management of prediabetes to prevent its progression to diabetes. Tseng et al. (2019) conducted a study in the United States, which showed that only 36% of PCPs referred their patients to Diabetic Lifestyle Programs (DLPs) for lifestyle change programs as the initial management strategy [6]. In Saudi Arabia, the knowledge and practice of PCPs were assessed, and the results showed that there are knowledge gaps, which require professional development for PCPs [7]. Although PCPs acknowledged the significance of the problem, a considerable number of them reported that they were not sufficiently confident in their ability to treat prediabetes adequately [8]. Moreover, an integrative review done on nutrition care for prediabetes revealed that there are significant gaps in KAP between patients and healthcare providers. It noted the need to improve collaboration and education to improve the quality of nutrition for prediabetes [9].

By establishing a clear understanding of the existing shortcomings and opportunities, healthcare authorities may design continuing medical education (CME) activities and materials to enhance PCP competence in prediabetes management. Strengthening the KAP of PCPs toward prediabetes will also be helpful for patients, due to the early diagnosis and effective treatment of this condition. Competent and well-equipped PCPs can facilitate the patient’s understanding of lifestyle changes and the need to adhere to a prescribed management plan to reduce T2DM occurrence and its complications. The aim of this study was to evaluate the level of awareness, perception, and management of prediabetes among the PCPs in Qassim, Saudi Arabia.

## Materials and methods

Study design and setting

A cross-sectional study was conducted among PCPs from February to April 2025 in the primary healthcare centers in Qassim, Saudi Arabia.

Target population: The target population in this study was the PCPs in primary healthcare centers in Qassim province.

Inclusion criteria: We included PCPs who were practicing in public or private sector primary healthcare centers in Qassim province.

Exclusion criteria: PCPs who are on leave or not practicing during the data collection period. We will also exclude interns and undergraduate students currently on rotation in primary care.

Sample size and sampling technique

Given that the population of PCPs is finite in the region (359) [10]. We conducted a census enumeration of all the eligible participants in our study. After administrative approvals, the primary healthcare centers were visited by the principal investigator, and potential participants were informed about the study and assessed for eligibility. Those who met the eligibility criteria were invited to participate in the study, and informed consent was obtained.

Data collection tools

The survey was conducted through a structured, self-completed questionnaire that was constructed based on previous research and validated questionnaires used in similar studies [3,7,11]. The questionnaire was designed to assess the following domains: demographics, knowledge, attitudes, and practices related to prediabetes (Appendices). The demographic part of the survey collected data on the age, gender, years of experience, and specialty of the respondents. For example, they were categorized by the type of practice (family medicine, general practice, etc.) and the place of work (urban or rural). Knowledge questions focused on the respondents’ understanding of prediabetes, risk factors for developing this condition, diagnostic criteria (e.g., fasting plasma glucose and glycated hemoglobin (HbA1c)), and management strategies (including dietary and pharmacological interventions). The attitudes part of the questionnaire addressed the perceived role and utility of screening and treatment of prediabetes among PCPs, their level of self-efficacy when it comes to managing prediabetes, and perceived barriers to effective management of prediabetes. The practices portion determined how often PCPs are screening for prediabetes, what diagnostic tools they use, treatment approaches, and how they refer patients to lifestyle intervention programs.

Data collection procedures

The questionnaires were delivered to PCPs working in both urban and rural health facilities. The questionnaire was administered over a period of three months in order to collect data. In each healthcare center, there was a coordinator who was responsible for the administration of questionnaires. Follow-up was made to increase the response rate. The remaining non-respondents were followed up by phone calls and emails.

Pilot study

Pilot testing of the questionnaire was done on 15 selected PCPs from an area other than the study setting to check the understandability, relevance, and inclusiveness of the questionnaire items. The data gathered from the pre-test were used to improve the questionnaire, as it helped in determining the right questions to be asked to get the data required for the study.

Data analysis

The questionnaires were collected from the respondents, and their validity was checked to ensure that they were completed. Data were keyed into a password-protected database to ensure privacy and data safety. Analyses were carried out using Statistical Package for the Social Sciences (SPSS) version 25.0 (IBM Corp., Armonk, NY, US). To analyze demographic data and scores for KAP, descriptive statistics were used. Mean and median for continuous variables were reported as measures of central tendency while standard deviation and inter-quartile range were used as measures of dispersion. Descriptive statistics were used in presenting the results of the categorical variables; these include frequencies and percentages. KAP scores were computed from the participant responses and summed up to get a composite score. In each domain, there was a maximum possible score, and all scores were presented as means with standard deviations. To compare the domain score of KAP, an independent sample t-test was applied for gender, specialty, and location of practice. The Pearson correlation coefficient was calculated for the association between quantitative variables such as age and years of experience with KAP scores. A p-value of less than 0.05 was considered statistically significant.

Ethical considerations

The study was carried out in compliance with the principles of the Helsinki Declaration, and ethical approval was sought from the Qassim Regional Bioethics Committee (approval number 607/46/8260). In addition, permission from the Ministry of Health and any other relevant health departments was sought to conduct the study in primary health care centers. Informed consent was sought from the primary healthcare providers to participate in the study. The consent form contained details regarding the study’s aim and purpose, the strategies employed in the study, the risks involved in the study, and the possible benefits of the study. To ensure the participants provided accurate information, they were informed that their responses would remain anonymous and would not be used for any purpose other than for research.

## Results

A total of 163 out of 344 eligible PCS (response rate: 47.4%) working in the Qassim region participated in the study. The mean age of the participants was 35.2 years (SD = 9.44), with nearly equal gender distribution; 51.5% were male (n = 84). Participants had an average of 7.6 years of professional experience (SD = 7.87). Regarding specialties, 56.4% (n = 92) were family physicians (having formal training in Family Medicine) and 43.6% (n = 71) were General Practitioners. The majority were working in urban settings (87.7%, n = 143), with 12.3% (n = 20) in rural areas. Most were employed in government health facilities (98.8%, n = 161), while a small minority worked in private settings (1.2%, n = 2) (Table 1).

**Table 1:** Sociodemographic and professional characteristics of primary care physicians (n = 163)

Variable	% (n)
Age	
Mean (SD)	35.2 (9.44)
Gender	
Female	48.5 (79)
Male	51.5 (84)
Years of experience	
Mean (SD)	7.6 (7.87)
Specialty	
Family Medicine	56.4 (92)
General Practitioner	43.6 (71)
Location of Practice	
Urban	87.7 (143)
Rural	12.3 (20)
Type of Healthcare Facility	
Government	98.8 (161)
Private	1.2 (2)

Knowledge assessment showed that most participants correctly identified major risk factors for prediabetes. All respondents recognized overweight/obesity as a risk factor, while nearly all also acknowledged family history (99.4%, n = 162), physical inactivity (96.9%, n = 158), and a history of gestational diabetes (96.3% n = 157). However, only 38.0% (62) and 41.1% (67) identified hypertension and dyslipidemia, respectively, as contributing risk factors. Knowledge of diagnostic criteria was similarly high, with 100% (163) identifying HbA1c levels of 5.7-6.4% (39 - 46 mmol/mol) and 96.9% (158) recognizing fasting plasma glucose levels of 100-125 mg/dL (5.556 - 6.944 mmol/L). Fewer respondents identified random plasma glucose levels (60.1%, n = 98) and oral glucose tolerance test (OGTT) values (79.1%, n = 129) within the diagnostic range. In terms of management, all respondents endorsed lifestyle modification, and 98.2% indicated metformin as a treatment option. Bariatric surgery was acknowledged by 70.6% (115), while only a small proportion recognized the use of statins (16.6%, n = 27) or antihypertensive medications (15.3%, n = 25). For follow-up intervals to monitor the progression to diabetes, 69.3% (113) suggested annual reviews, with smaller percentages opting for every six months (22.1%, n = 36) or every three months (8.6%, n = 14). Just over half of the respondents (52.8%, n = 86) reported familiarity with the American Diabetes Association (ADA) management guidelines (Table 2).

**Table 2:** Knowledge of primary care physicians about prediabetes (n = 163) OGTT: oral glucose tolerance test; ADA: American Diabetes Association

Variable	% (n)
Knowledge about risk factors: (Yes)	
Age equal to or more than 35	78.5 (128)
Family history of diabetes	99.4 (162)
Overweight/Obesity	100 (163)
Physical inactivity	96.9 (158)
Hypertension	38.0 (62)
Dyslipidemia	41.1 (67)
History of gestational diabetes	96.3 (157)
Polycystic ovary syndrome (PCOS)	92.0 (150)
Knowledge about diagnostic criteria: (Yes)	
Fasting plasma glucose (100-125 mg/dL)	96.9 (158)
2-hour plasma glucose during OGTT (140-199 mg/dL)	79.1 (129)
HbA1c (5.7-6.4%)	100 (163)
Random plasma glucose (140-199 mg/dL)	60.1 (98)
Knowledge about management: (Yes)	
Lifestyle modifications (diet and exercise)	100 (163)
Metformin	98.2 (160)
Bariatric surgery	70.6 (115)
Statins	16.6 (27)
Antihypertensive medications	15.3 (25)
Knowledge about follow-up for progression to diabetes	
Every 3 months	8.6 (14)
Every 6 months	22.1 (36)
Annually	69.3 (113)
Every 2 years	0 (0)
Knowledge about ADA management guidelines	
Yes	52.8 (86)
No	47.2 (77)

Attitudes toward prediabetes were generally positive. Screening was considered either very important (52.1%) or important (47.2%, n = 77) by nearly all participants. Most felt confident in their ability to manage prediabetes, with 25.2% (41) reporting that they were very confident and 52.8% (86) were confident. Nearly all participants (98.8%, n = 161) agreed or strongly agreed that lifestyle modification is the most effective intervention. The most commonly cited barriers to effective management included patient non-compliance (97.5%, n = 159), lack of awareness (88.3%, n = 144), and lack of time (84.7%, n = 138). Fewer physicians reported a lack of resources (53.4%, n = 87) and insufficient training (54.6%, n = 89) as challenges. When considering referral to diabetes prevention programs, 48.5% (79) indicated they were likely and 22.1% (36) very likely to do so (Table 3).

**Table 3:** Attitudes of primary care physicians about prediabetes (n = 163)

Variable	% (n)
Perceived importance of screening	
Very important	52.1 (85)
Important	47.2 (77)
Neutral	0.6 (1)
Unimportant	0 (0)
Very unimportant	0 (0)
Confidence to manage	
Very confident	25.2 (41)
Confident	52.8 (86)
Neutral	20.2 (33)
Not confident	1.8 (3)
Not confident at all	0 (0)
Lifestyle modification is the most effective intervention in management	
Strongly agree	58.3 (95)
Agree	40.5 (66)
Neutral	1.2 (2)
Disagree	0 (0)
Strongly disagree	0 (0)
Perceived barriers for effective management: (Yes)	
Lack of time	84.7 (138)
Lack of resources	53.4 (87)
Patient non-compliance	97.5 (159)
Insufficient training	54.6 (89)
Lack of awareness	88.3 (144)
Referring patients with prediabetes to a prevention program	
Very likely	22.1 (36)
Likely	48.5 (79)
Neutral	14.1 (23)
Unlikely	12.3 (20)
Very unlikely	3.1 (5)

In terms of clinical practice, 81% (132) of physicians reported screening for prediabetes, either always (39.9%, n = 65) or often (41.1%, n = 67). The most utilized diagnostic methods were HbA1c (99.4%, n = 162) and fasting plasma glucose (98.2%, n = 162), with OGTT and random glucose being less commonly used. All participants were advised on lifestyle modification, and 92% (150) were prescribed metformin. Additional practices included referrals to dietitians (76.7%, n = 125), enrolment in diabetes prevention programs (57.1%, n = 93), and follow-up with regular blood tests (95.1%, n = 155). Most physicians reported following up with patients every six months (46%, n = 75) or every three months (40.5%, n = 66). Only 16.6% (27) provided written educational materials to patients. In terms of resource availability, most rated them as fair (51.5%, n =84) or good (33.7%, n = 55), while 8.6% (14) considered them excellent. Most participants believed that additional resources, such as CME training (93.9%, n = 153), access to dietitians (96.9%, n = 158), patient education materials (97.5%, n = 159), and support groups (87.1%, n = 142) would improve management (Table 4).

**Table 4:** Practice of primary care physicians related to prediabetes (n = 163)

Variable	% (n)
Frequency of screening for prediabetes	
Always	39.9 (65)
Often	41.1 (67)
Sometimes	16.6 (27)
Rarely	2.5 (4)
Never	0 (0)
Methods to diagnose prediabetes: (Yes)	
Fasting plasma glucose test	98.2 (160)
Oral glucose tolerance test (OGTT)	4.6 (8)
HbA1c test	99.4 (162)
Random plasma glucose test	17.8 (29)
Management of Prediabetes: (Yes)	
Advise lifestyle modifications	100 (163)
Prescribe metformin	92 (150)
Refer to a dietitian	76.7 (125)
Enroll in a diabetes prevention program	57.1 (93)
Follow up with regular blood tests	95.1 (155)
Follow up patients diagnosed with prediabetes	
Every 3 months	40.5 (66)
Every 6 months	46.0 (75)
Annually	13.5 (22)
Only if symptomatic	0 (0)
Provide written educational materials to patients	
Yes	16.6 (27)
No	83.4 (136)
Availability of resources for management	
Excellent	8.6 (14)
Good	33.7 (55)
Fair	51.5 (84)
Poor	6.1 (10)
Very poor	0 (0)
Additional resources help in management: (Yes)	
More training/CME programs	93.9 (153)
Access to dietitians and nutritionists	96.9 (158)
Patient education materials	97.5 (159)
Support groups for patients	87.1 (142)
Better screening tools	36.2 (59)

**Figure 1:**
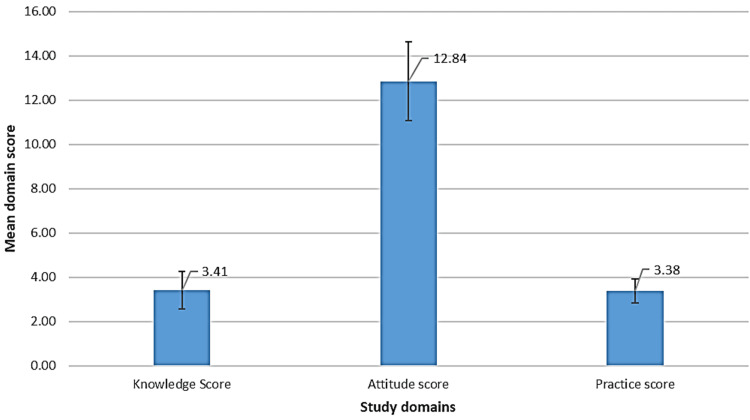
Mean prediabetes knowledge, attitude, and practice scores of PHC physicians PHC: primary health care

Figure 1 presents the mean prediabetes knowledge, attitude, and practice scores of primary health care (PHC) physicians. The mean knowledge score was 3.41 out of a maximum of 5, and the mean attitude score was 12.84 out of 16. The practice score was 3.38 out of 5.

Regarding continuing medical education (CME), only 20.2% (33) of respondents had attended a CME program on prediabetes in the past year. Among them, most attended one or two sessions. The perceived effectiveness of these CME sessions was high, with 81.25% (26) rating them as very effective or effective. The majority expressed interest in future CME topics such as the latest management guidelines (96.9%, n = 158), patient counseling techniques (90.8%, n = 148), case studies (90.2%, n = 147), and technology integration (93.9%, n = 153) (Table 5).

**Table 5:** Perceived needs and challenges in continuing medical education (CME) regarding prediabetes (n = 163)

Variable	% (n)
Participation in any CME programs last year	
Yes	20.2 (33)
No	79.8 (130)
Number of CME attended (n = 32)	
1 to 3	90.6 (29)
3 to 4	9.4 (3)
More than 4	0 (0)
Perceived effectiveness of CME programs: (n = 31)	
Very effective	35.5 (11)
Effective	48.4 (15)
Neutral	16.1 (5)
Ineffective	0 (0)
Very ineffective	0 (0)
Topics to be covered in the future CME programs: (Yes)	
Advances in diagnostic criteria	44.8 (73)
Latest management guidelines	96.9 (158)
Patient counseling techniques	90.8 (148)
Case studies and practical examples	90.2 (147)
Integration of technology in management	93.9 (153)
Significant challenges in management: (Yes)	
Lack of knowledge	39.3 (64)
Lack of screening facilities	20.2 (33)
Lack of patients' awareness about prediabetes	96.9 (158)
Patients non-compliance	96.3 (157)
Difficulty in patient follow-up	92 (150)
Difficulty in referral ( e.g. Nutritionist, etc.)	86.5 (141)
Financial difficulty	50.3 (82)
Strategies to improve the management of prediabetes: (Yes)	
CME programs focused on prediabetes	93.3 (152)
Implement routine screening protocols for high-risk individuals	98.8 (161)
Patient education about prediabetes	96.9 (158)
Schedule regular follow-ups (every 3–6 months) for lab monitoring and reinforcement of lifestyle changes.	99.4 (162)
Multidisciplinary Approach and easy referral	96.3 (157)
Better insurance coverage	96.3 (157)

Challenges in prediabetes management included lack of patient awareness (96.9%, n = 158), non-compliance (96.3%, n = 157), follow-up difficulties (92%, n = 150), referral issues (86.5%, n = 141), and financial barriers (50.3%, n = 82). To address these issues, participants recommended routine screening protocols for high-risk individuals (98.8%, n = 161), patient education initiatives (96.9%, n = 158), scheduled follow-ups (99.4%, n = 162), and multidisciplinary collaboration with easier referrals (96.3%, n = 157). Better insurance coverage and CME-focused strategies were also considered crucial by 96.3% (157) and 93.3% (153) of participants, respectively (Table 5).

Table 6 presents the relationship of socio-demographic and professional characteristics with knowledge, attitude, and practice scores. There was no significant relationship between age, gender, and years of experience. Family physicians had a higher knowledge score (3.84/5) as compared to general practitioners (2.85/5). Similarly, urban practitioners have higher scores (3.52/5) as compared to rural practitioners (2.65/5). Regarding attitude, age and years of experience had a significant but weak correlation with the attitude score. No significant relationship of attitude was found with gender and location of practice. Family medicine physicians had higher attitude scores than general practitioners. In the domain of practice, there was a significant but weak correlation between the practice score and age and years of experience. Gender, specialty, and location of practice were not significantly associated with practice.

**Table 6:** Association of socio-demographic and professional characteristics with knowledge, attitude, and practice score related to prediabetes (n = 163) GP: general practitioner

	Knowledge		Attitude		Practice	
Variable	Mean (SD)	p-value	Mean (SD)	p-value	Mean (SD)	p-value
Age						
Correlation coefficient	0.057	0.469	0.227	0.004	0.188	0.016
Gender						
Female	3.4 (0.85)	NA	12.7 (1.87)	NA	3.4 (0.49)	NA
Male	3.4 (0.84)	0.93	12.9 (1.69)	0.363	3.4 (0.60)	0.983
Years of experience						
Correlation coefficient	0.013	0.87	0.202	0.01	0.186	0.017
Specialty						
Family Medicine	3.84 (0.70)	NA	13.2 (1.73)	NA	3.43 (0.55)	NA
GP	2.85 (0.70)	<0.001	12.4 (1.75)	0.004	3.30 (0.52)	0.14
Location of practice						
Urban	3.52 (0.82)	NA	12.88 (1.71)	NA	3.40 (0.52)	NA
Rural	2.65 (0.58)	<0.001	12.55 (2.30)	0.439	3.20 (0.65)	0.13

## Discussion

This study aimed to assess the KAP of PCPs toward prediabetes in Qassim, Saudi Arabia. The findings reveal a concerning gap in both knowledge and the practical application of prediabetes management, despite generally positive attitudes among physicians.

Our findings are consistent with previous research conducted in Saudi Arabia. For instance, a 2022 study among PCPs in Riyadh revealed that only half of the respondents correctly identified the diagnostic thresholds for fasting plasma glucose and HbA1c levels in prediabetes [7]. A study from the United States also reported that only 41% and 21% of physicians were able to correctly identify the diagnostic criteria of prediabetes based on HbA1c and fasting blood glucose, respectively [11]. Similarly, a study among family medicine residents across Saudi Arabia showed an insufficient application of evidence-based screening guidelines [12]. These parallels suggest that knowledge gaps are not unique to Qassim but reflect a broader national challenge that requires the inclusion of this topic in the training curriculum and CME planning.

Regarding the attitudes of PCPs toward prediabetes screening, the results of the current study in Qassim are in line with national trends [7]. A systematic review of studies among various healthcare providers also reported a positive attitude [13]. The study found that while physicians generally had a favorable attitude toward managing prediabetes, this did not translate into adequate practice. For example, only 50% could correctly identify diagnostic thresholds, and there were significant gaps in routine screening and initiation of treatment. Similarly, our study found that although attitudes were largely positive, particularly among Family Physicians, there was a weak and inconsistent correlation with actual clinical practice. This reinforces the attitude-practice gap and suggests that positive perception alone is insufficient without system-level reinforcement and clinical integration.

A recent cross-sectional study in Central China by Pi et al. [3] found that while most (85.8%) PCPs had positive attitudes, their knowledge (54.7%) and practice (32.6%) scores were suboptimal. Specifically, only 54.7% achieved optimal knowledge scores, and less than one-third demonstrated adequate practice behaviors. Similarly, a systematic review also reported poor knowledge and varying practices among healthcare workers in different settings [13]. These results underscore the global nature of the issue and the critical need for targeted continuing medical education (CME).

Analysis of the relationship between socio-demographic and professional characteristics and KAP scores yielded important insights. Notably, there was no significant relationship between age, gender, and years of experience with knowledge scores. Similar were findings in a study from Sudan [4]. In contrast, a study from China reported higher knowledge and practice scores among female primary care providers as compared to males [3]. However, family physicians demonstrated significantly higher knowledge scores (3.84/5) as compared to general practitioners (2.85/5). In addition, physicians practicing in urban settings scored higher (3.52/5) than those in rural areas (2.65/5). This aligns with a Chinese study where providers in township facilities had better knowledge and attitude scores as compared to those in villages [3]. These findings indicate possible disparities in access to clinical resources or educational opportunities.

In the attitude domain, age and years of experience showed a significant but weak positive correlation with attitude scores. No significant relationship was found between attitude scores and gender or location of practice. On the other hand, gender had no association with attitude, but those in rural settings had lower attitude scores in China [3]. Nevertheless, family medicine physicians maintained higher attitude scores than general practitioners. This finding is similar to the report from China [3]. This suggests the influence of specialized training on perceptions and clinical orientation toward prediabetes care.

For practice scores, there was also a significant but weak correlation with age (r = 0.188) and years of experience (r = 0.186), while gender, specialty, and practice location were not significantly associated. This indicates that, despite positive attitudes and increased knowledge in certain subgroups, these may not consistently translate into improved clinical practices without systemic support or structured interventions.

Despite generally favorable attitudes toward the importance of prediabetes management, this study observed a notable disconnect between attitude and clinical practice. This phenomenon has been observed in various KAP studies, where clinicians express concern about a condition but fail to implement evidence-based actions. Contributing factors may include limited consultation time, lack of CME participation, and low patient compliance [3,14,15].

In our study, out of 163 respondents, only 33 physicians (20.2%) reported participation in continuing medical education (CME) related to diabetes, while 130 (79.8%) had not participated in any recent CME. This participation rate is considerably lower than findings from other Saudi-based studies [16,17]. For instance, in a study, 39 out of 51 (76.5%) physicians attended diabetes CME sessions during the last five years, which significantly improved their KAP scores [16]. Similarly, in a cross-sectional study from Abha, Saudi Arabia, 65.5% of 267 healthcare professionals had attended at least 1 CME session in the past year [17]. A study from Sudan also reported that about 63% of the physicians had attended educational activities related to prediabetes [4]. This finding underscores a concerning gap in CME engagement among Qassim’s PCPs, which may contribute to the relatively lower knowledge and practice scores observed in our cohort. We think that this is because Qassim’s PCPs cannot get a release from their duties to attend these kinds of CME programs, and there is a lack of locally held CME sessions.

Regarding the challenges, PCPs reported several significant challenges in managing prediabetes. The most common barrier was a lack of patients' awareness about prediabetes, cited by 96.9% (158) of participants. This was followed by patients’ non-compliance with management plans (96.3%, 157), difficulty in patient follow-up (92%, 150), and difficulty in referral processes such as to a nutritionist (86.5%, 141). Financial difficulty was reported by 50.3% (82), and 39.3% (64) identified the lack of knowledge as a barrier. The least reported challenge was a lack of screening facilities, noted by 20.2% (33) of participants. These findings overlap with the various challenges reported in a systematic review [13].

To address these challenges, several targeted solutions are recommended. Due to the lack of patient awareness, public health campaigns and community-based education initiatives should be launched to inform the population about prediabetes risks and prevention. To combat non-compliance, implementing specific counseling and involving social workers in primary care may improve adherence. Enhancing follow-up can be achieved through telemedicine, automated appointment reminders, and better integration of electronic health records with higher-level facilities. Addressing financial difficulties may require policies that support indirect patient costs through transportation vouchers or workplace health screenings. The lack of physician knowledge underscores the need for mandatory, accessible CME programs. Finally, improving diagnostic infrastructure will mitigate issues related to limited screening facilities, particularly in under-resourced centers.

This study has some limitations. The cross-sectional design precludes causal inferences. Self-reported data may be subject to social desirability and recall biases. Also, the findings are limited to Qassim and may not be generalizable to other regions in Saudi Arabia. Nonetheless, this study adds to the scarce literature on the knowledge and practices of primary care physicians regarding prediabetes and provides important insights into the issue.

## Conclusions

In conclusion, while primary care physicians in Qassim demonstrate a positive outlook (12.84/16) toward prediabetes management, significant deficiencies in knowledge (3.42/5) and practices (3.32/5) persist. Differences by specialty and location are also prevalent. These findings highlight the urgent need for structured interventions, including mandatory CME focused on diabetes prevention and management and the integration of prediabetes modules into routine primary care workflows. The development of region-specific clinical decision support tools, such as red flags in electronic health records, is also needed. Additionally, national health authorities may consider implementing regular audits and feedback mechanisms to reinforce adherence to prediabetes guidelines. Further research is also needed at the national level to produce generalized results.

## Appendices

Questionnaire

Section A: Demographic Information

1.     Age:__________Years

2.     Gender:

·       Male

·       Female

3.     Years of experience: __________Years

4.     Specialty:

·       Family Medicine

·       General Practice

·       Internal Medicine

·       Endocrinology

·       Other (Please specify): _______________

5.    Location of Practice:

·       Urban

·       Rural

6.    Type of Healthcare Facility:

·       Government

·       Private

·       Other (Please specify): _______________

Section B: Knowledge of Prediabetes

7.    What are the primary risk factors for prediabetes? (Select all that apply)

·       Age equal or more than 35

·       Family history of diabetes

·       Overweight/Obesity

·       Physical inactivity

·       Hypertension

·       Dyslipidemia

·       History of gestational diabetes

·       Polycystic ovary syndrome (PCOS)

8.    What are the diagnostic criteria for prediabetes? (Select all that apply)

·       Fasting plasma glucose 100-125 mg/dL

·       2-hour plasma glucose during OGTT 140-199 mg/dL

·       HbA1c 5.7-6.4%

·       Random plasma glucose 140-199 mg/dL

9.    Which of the following interventions are recommended for managing prediabetes? (Select all that apply)

·       Lifestyle modifications (diet and exercise)

·       Metformin

·       Bariatric surgery

·       Statins

·       Antihypertensive medications

10.   How often should patients with prediabetes be monitored for progression to diabetes?

·       Every 3 months

·       Every 6 months

·       Annually

·       Every 2 years

11.    Are you familiar with the American Diabetes Association (ADA) guidelines for the management of prediabetes?

·       Yes

·       No

Section C: Attitudes Toward Prediabetes Management

12.   How important do you think it is to screen for prediabetes in patients with risk factors?

·       Very important

·       Important

·       Neutral

·       Unimportant

·       Very unimportant

13.   How confident do you feel in your ability to manage patients with prediabetes?

·       Very confident

·       Confident

·       Neutral

·       Not confident

·       Not confident at all

14.   To what extent do you agree with the following statement: "Lifestyle modification is the most effective intervention for managing prediabetes."

·       Strongly agree

·       Agree

·       Neutral

·       Disagree

·       Strongly disagree

15.   What do you perceive as the main barriers to effective prediabetes management in your practice? (Select all that apply)

·       Lack of time

·       Lack of resources

·       Patient non-compliance

·       Insufficient training

·       Lack of awareness

·       Other (Please specify): _______________

16.   How likely are you to refer patients with prediabetes to a diabetes prevention program?

·       Very likely

·       Likely

·       Neutral

·       Unlikely

·       Very unlikely

Section D: Practices Related to Prediabetes Management

17.    How frequently do you screen for prediabetes in patients with risk factors?

·       Always

·       Often

·       Sometimes

·       Rarely

·       Never

18.   What methods do you use to diagnose prediabetes in your practice? (Select all that apply)

·       Fasting plasma glucose test

·       Oral glucose tolerance test (OGTT)

·       HbA1c test

·       Random plasma glucose test

19.   How do you typically manage a patient diagnosed with prediabetes? (Select all that apply)

·       Advise lifestyle modifications

·       Prescribe metformin

·       Refer to a dietitian

·       Enrol in a diabetes prevention program

·       Follow up with regular blood tests

20.   How often do you follow up with patients diagnosed with prediabetes?

·       Every 3 months

·       Every 6 months

·       Annually

·       Only if symptomatic

21.   Do you provide written educational materials to patients with prediabetes?

·       Yes

·       No

22.  How do you rate the availability of resources for managing prediabetes in your healthcare facility?

·       Excellent

·       Good

·       Fair

·       Poor

·       Very poor

23.   What additional resources or support would help you manage prediabetes more effectively? (Select all that apply)

·       More training/CME programs

·       Access to dietitians and nutritionists

·       Patient education materials

·       Support groups for patients

·       Better screening tools

·       Other (Please specify): _______________

Section E: Continuing Medical Education and Training

24.   Have you participated in any continuing medical education (CME) programs focused on prediabetes in the last year?

·       Yes

·       No

25.   If yes, how many CME programs on prediabetes have you attended in the last year?

·       1-2

·       3-4

·       More than 4

26.   How effective do you find CME programs in improving your knowledge and practice regarding prediabetes?

·       Very effective

·       Effective

·       Neutral

·       Ineffective

·       Very ineffective

27.   What topics would you like to see covered in future CME programs on prediabetes? (Select all that apply)

·       Advances in diagnostic criteria

·       Latest management guidelines

·       Patient counseling techniques

·       Case studies and practical examples

·       Integration of technology in management

·       Other (Please specify): _______________

Section F: Perceived challenges and solutions

28.   What do you believe are the most significant challenges in managing prediabetes in your practice?

·       Lack of knowledge

·       Lack of screening facilities

·       Lack of patients' awareness about prediabetes

·       Patients’ non-compliance

·       Difficulty in patients’ follow-up

·       Difficulty in referral (e.g. Nutritionist, etc.)

·       Financial difficulty

29.   In your opinion, what strategies could improve the management of prediabetes in primary care settings?

·       Continuing medical education (CME) programs focused on prediabetes

·       Availability of screening facilities and implement routine screening protocols for high-risk individuals

·       Patient education & counseling about prediabetes

·       Schedule regular follow-ups (every 3-6 months) for lab monitoring and reinforcement of lifestyle changes

·       Multidisciplinary Approach and easy referral: Involving dietitians, diabetes educators, and health coaches

·       Promoting better insurance coverage for preventive care and diabetes screening
